# Mutations in the *Plasmodium falciparum cytochrome b *gene are associated with delayed parasite recrudescence in malaria patients treated with atovaquone-proguanil

**DOI:** 10.1186/1475-2875-7-240

**Published:** 2008-11-20

**Authors:** Colin J Sutherland, Matt Laundy, Nicholas Price, Martina Burke, Quinton L Fivelman, Geoffrey Pasvol, John L Klein, Peter L Chiodini

**Affiliations:** 1 Department of Clinical Parasitology, Hospital for Tropical Diseases, Mortimer Market, Capper St, London, WC1E 6AU, UK; 2 HPA Malaria Reference Laboratory, London School of Hygiene & Tropical Medicine, London, UK; 3 Infection and Immunology Delivery Unit, St. Thomas' Hospital, London, UK; 4 Imperial College London, Lister Unit, Northwick Park Hospital, London, UK

## Abstract

**Background:**

Fixed-dose combination antimalarial drugs have played an increasingly important role in the treatment and chemoprophylaxis of falciparum malaria since the worldwide failure of monotherapy with chloroquine. Atovaquone-proguanil is one such combination drug used both for prophylaxis in travellers, and for treatment of acute malaria cases in European hospitals and clinics.

**Methods:**

A series of eight atovaquone-proguanil treatment failures and two prophylaxis breakthroughs from four UK hospitals from 2004–2008 were analysed for evidence of mutations in the *pfcyt-b *gene, previously found to be associated with failure of the atovaquone component.

**Results:**

Parasites carrying *pfcyt-b *mutations were found in five falciparum malaria patients with recrudescent parasitaemia occurring weeks after apparently successful treatment of a primary infection with atovaquone-proguanil. Four of these cases carried parasites with the Tyr268Cys mutation in *pfcyt-b*, previously reported in two French patients with malaria. In contrast, mutations in *pfcyt-b *were not found in three patients treated with atovaquone-proguanil who exhibited delayed clearance of the primary infection, nor in two returning travellers with malaria who had used the combination for prophylaxis. Using current and previously published data, mean time to recrudescence of parasites carrying *pfcytb *codon 268 mutations was estimated as 28.0 days after treatment (95% C.I. 23.0 – 33.0 days), whereas treatment failures without codon 268 mutations received rescue treatment an average of 4.71 days after initial AP treatment (95% C.I. 1.76 – 7.67 days).

**Conclusion:**

Genetically-determined parasite resistance to atovaquone is associated with delayed recrudescence of resistant parasites three weeks or more after initial clearance of parasitaemia by atovaquone/proguanil therapy. The 268-Cys allele of *pfcyt-b *may have been overlooked in previous studies of atovaquone-proguanil treatment failure as it is not detected by current RFLP methods.

## Background

The hydroxynaphthoquinone compound atovaquone was developed in the 1990s as an anti-protozoal drug, with demonstrated activity against cytochrome b in pathogen mitochondria. High rates of post-treatment recrudescence in patients treated with atovaquone alone for *Plasmodium falciparum *malaria [1] may be attributable to *de novo *mutations in the parasite's *pfcyt-b *gene that arise from increased oxidative damage to mitochondrial DNA generated by the action of atovaquone [2]. This led to deployment of atovaquone in combination with other antimalarials, and in particular in a highly synergistic fixed combination with proguanil as atovaquone/proguanil (AP; Malarone^®^).

AP is widely used for malaria prophylaxis in travellers, and for treatment of clinical malaria cases in high income countries. It is not used as therapy in sub-Saharan Africa due to its high cost, but there are reports of *pfcyt-b *mutations in African parasite isolates, mostly following atovaquone monotherapy or treatment with AP [3-6]. Malaria patients failing AP therapy may carry the resistance-associated *pfcyt-b *mutations Tyr268Ser or Tyr268Asn [3,7], although treatment failure is also observed in the absence of these mutations [8]. Such parasites may have intermediate *in vitro *sensitivity to atovaquone compared to that of parasites carrying codon 268 mutations in *pfcyt-b *[5].

DNA sequencing analysis was performed on *P. falciparum cyt-b *genes from malaria patients with recurrent parasitaemia following treatment with AP in four UK settings, or following use of AP prophylaxis in a malaria endemic area. The relevance of these results for malaria treatment policy in the UK is discussed.

## Methods

Parasites were isolated from three categories of patients with falciparum malaria: those whose clinical and parasitological response to AP was delayed in the first three days following AP treatment, those with microscopically confirmed recrudescent parasitaemia following initially successful treatment with AP, and three cases where AP had been taken as prophylaxis during travel in an area of malaria endemicity. Genotyping of the *pfcyt-b *gene was carried out as part of the surveillance remit of the Malaria Reference Laboratory (MRL), and performed either at the Department of Clinical Parasitology, Hospital for Tropical Diseases (HTD) or at the LSHTM Malaria Reference Laboratory (MRL), using identical PCR and sequencing protocols. The MRL is mandated by the UK Health Protection Agency to provide molecular surveillance in the form of genetic typing of *P. falciparum *for drug resistance-associated alleles, to assist in development of national policy on prevention and treatment of imported malaria. The work described was carried out under this mandate. All patient identifiers have been removed from this report.

A 939 bp portion of the *pfcyt-b *gene was amplified using primers cyt-b1 (5'CTC TAT TAA TTT AGT TAA AGC ACA 3') and cyt-b2 (5' ACA GAA TAA TCT CTA GCA CC 3') by conventional PCR with standard regents. Reaction conditions were used as follows: samples were heated initially at 93°C for 10 minutes and then at 93°C for 50 s, 45°C for 50 s and 70°C for 1 min over 50 cycles. PCR products were purified by fractionation on a 1% agarose gel and elution with the MinElute Gel Extraction Kit (Qiagen Ltd, Crawley UK).

PCR products were sequenced using the ABI Prism Big Dye Terminator kit, using the amplification primers and a third primer, cyt-b7 (5'-CAA TTA CTA AAC CAG CTG G-3'), to prime the sequencing reactions. Electropherogram data were edited, collated and analysed using Chromas software (Technelysium, Australia). All DNA sequences were confirmed by at least two independent sequence reads.

## Results

### Patients

The sequence surrounding codon 268 of the *pfcyt-b *gene was determined for each suspected atovaquone-resistant parasite isolate. These were obtained from the following patients:

*Patient 1 *was a 29 year old male, admitted in October 2004 with uncomplicated falciparum malaria (parasitaemia 1.1%). Although ethnically of African origin, he was born and resident in the UK. Approximately six weeks prior, this patient had been treated for falciparum malaria acquired in Sierra Leone with a full course of AP (four tablets daily for three days). His symptoms resolved at that time, although parasitological confirmation of cure was not obtained. The recrudescent infection was treated with quinine/Fansidar^® ^(sulfadoxine/pyrimethamine). Parasite DNA was extracted from two sequential peripheral blood samples taken nine hours apart at the time of his recrudescent infection.

*Patient 2 *was a 44 year-old male who presented in August 2004 with 1% *P. falciparum *parasitaemia after travel in Nigeria, where an unspecified antimalarial injection had been received some weeks earlier. His previous history of exposure and thus immune status with respect to malaria is unknown. Parasitaemia rose under treatment with AP to 4% with continuing symptoms on day 3 of admission. Treatment was then switched to intravenous quinine. DNA was extracted from a single pre-treatment sample.

*Patient 3 *was a 60-year old female treated with a full course of AP for falciparum malaria in July 2004 following a trip to Nigeria. She was a UK resident who had been brought up in Nigeria. There were no clinical features of severe disease, although initial parasitaemia was 2.5%. Sixty-eight hours following the start of her course of AP she returned with persisting symptoms and a parasitaemia of 0.1%. She was commenced on a course of quinine therapy and rapidly improved. A blood film taken the following day was parasite negative.

*Patient 4*, a five-year old boy, presented in October 2004 with a 0.1% *P. falciparum *parasitaemia following a trip to Nigeria. Although ethnically African, he had lived all his life in the UK. He made a prompt and unremarkable clinical recovery following a full course of AP. Symptoms recurred 23 days later and the child re-presented after a further two days with a 0.3% parasitaemia. DNA was extracted from a peripheral blood sample taken at the time of his recrudescent infection.

*Patient 5*, a 21-year old male resident in London for seven years but having spent his childhood in Sierra Leone, presented with a *P. falciparum *parasitaemia of 0.8% in September 2006 following a visit to his country of origin. He reported full adherence to AP prophylaxis (one tablet daily).

*Patient 6 *was a 56-year old female who presented to hospital with microscopy-confirmed falciparum malaria within a few days of returning to her home in London from Nigeria in August 2006. The patient informed hospital staff of a known allergy to quinine, suggesting suspected malaria infections had been treated in the past. She was treated with a full course of AP. Persisting parasitaemia at 1% after an additional 4th day of AP treatment precipitated a change to quinine with antihistamine cover, which cleared the parasites. A blood sample was sent for DNA sequencing analysis.

*Patient 7 *was a 30-year old male born and brought up in Uganda but was a UK resident at the time of the malaria episode. He was diagnosed with falciparum malaria in Switzerland in September 2007 after travel to Uganda, and reported full adherence to a three day therapeutic course of AP. Parasite clearance was not confirmed microscopically. He presented again in London 21 days after treatment with 0.2% *P. falciparum *parasitaemia and was treated with a course of quinine. DNA was extracted for analysis from a blood sample collected at the time of his recrudescent infection.

*Patient 8 *was a 36-year old male presenting with uncomplicated falciparum malaria, parasitaemia less than 0.1%, in June 2008 after visiting Uganda and Kenya without taking malaria prophylaxis. This individual was born and brought up in Uganda, but had been resident in the UK for 21 years. He was treated with a full 3-day course of AP and resolution of symptoms was reported over the following two days. His symptoms returned 21 days later and a repeat blood film confirmed recrudescent *P. falciparum *parasitaemia of less than 0.1%. Parasite material was retrospectively retrieved from a Giemsa-stained thin film from the primary episode and compared with results obtained from the blood sample collected at the time of recrudescence.

*Patient 9 *was a 47-year old male presenting with uncomplicated falciparum malaria (parasitaemia < 0.1%) in July 2008 after visiting Sierra Leone. He reported good adherence to AP prophylaxis.

*Patient 10 *was a 47-year old British female who worked in a rural school in The Gambia from March 2008 to June 2008, and taking mefloquine prophylaxis for that period. On 30^th ^June, having discontinued chemoprophylaxis, she travelled to Senegal and within 3 days developed fever. She received a presumptive diagnosis of malaria, and took a 3 day course of AP from a back-up supply she carried with her, and reported that her symptoms resolved. This individual returned to the UK on the 8th of July. She took 1 further dose of mefloquine from her prophylaxis course on return to the UK. She attended her GP 4 weeks later with fatigue and headache, and a blood film was *P. falciparum *positive with a parasite count of 3%. The patient was treated successfully with quinine followed by doxycycline.

Sequencing data for the *pfcyt-b *locus are presented for these ten patients in Table 1.

**Table 1:** Description of patients and sequencing results.

**Patient #**	**Sample #**	**Sample Date**	**Hospital/GP**	**Geography**	**AP Use**	**Days since AP treatment**	***Pfcyt-b *aa268**
1	A (relapse)	October 2004	St Thomas'	Sierra Leone	Treatment	42	Cys
							
	B (relapse)	October 2004					Cys

2	A	September 2004	Northwick Pk	Nigeria	Treatment	2	Tyr

3	A	July 2004	St Thomas'	Nigeria	Treatment	3	Tyr

4	A	October 2004	St Thomas'	Nigeria	Treatment	25	Ser

5	A	Sept 2006	St Thomas'	Sierra Leone	Prophylaxis	N/A	Tyr

6	A	Aug 2006	Homerton/HTD	Nigeria	Treatment	4	Tyr

7	A	Sept 2007	St Thomas'	Uganda	Treatment	21	Cys

8	A (pre-treat)	June 2008	St Thomas'	Uganda/Kenya	Treatment	26	Tyr
							
	B (relapse)	July 2008					Cys

9	A	July 2008	St Thomas'	Sierra Leone	Prophylaxis	N/A	Tyr

10	A	August 08	Bristol	Gambia/Senegal	Treatment	32	Cys

**Figure 1:**
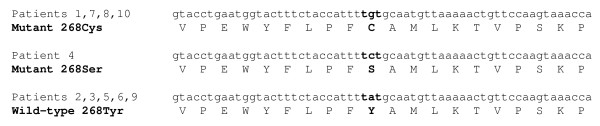
**DNA sequence encoding amino acids 259 – 279 of *pfcyt-b *in 10 *P. falciparum *isolates from UK malaria patients**. Patients 1, 7, 8 and 10 represent four instances of the recently confirmed 268-Cys genotype. Patients 2, 3, 5, 6 and 9 carry the wild-type genotype (268-Tyr), and patient 4 the previously reported atovaquone-resistant genotype (268-Ser).

Two different mutations at position 268 of the *pfcyt-b *gene were identified among the parasite isolates investigated: patient 4 carried parasites with the Tyr-268-Ser allele of *pfcyt-b *as previously described [3], whereas patient 1 (both samples A and B) and patients 7, 8 (July recrudescent sample only) and 10 harboured parasites with the Tyr-268-Cys allele (Figure 1) [5]. Each of these five patients had a recrudescent *P. falciparum *infection at least three weeks after a primary malaria episode which initially resolved with AP treatment. No cases harbouring the Tyr-268-Asn allele of *pfcyt-b *were identified, and no mutations were identified at any other codons of the *pfcyt-b *gene. During his first (June) episode of malaria, Patient 8 harboured parasites with the wild-type Tyr at codon 268 of *pfcyt-b*, but 26 days later only parasites with Cys encoded at position 268 were identified.

Patients 2, 3 and 6 exhibited delayed clearance of *P. falciparum *infections in the first 72 hours following initiation of AP treatment, precipitating a change in therapy (to quinine in each case). Each patient carried parasites with *pfcyt-b *genes that were wild-type at codon 268. Patients 5 and 9 reported good adherence to AP prophylaxis during visits to malaria endemic zones. *Plasmodium falciparum *parasites isolated from both of these patients were wild type at codon 268 of the *pfcyt-b *locus.

These results suggest that genetically determined resistance to atovaquone at codon 268 of *pfcyt-b *is associated with recrudescence of parasites at least 20 days after apparently successful AP therapy. To test this possibility, data from this series of eight patients for whom AP therapy failed were analysed together with ten such patients described by Musset *et al. *[5]. Prophylaxis failures (patients 5 and 9) were not included in the analysis. Seven of these 18 patients harboured wild-type parasites at the time of treatment failure, which occurred at a mean of 4.71 days after initial AP treatment (95% C.I. 1.76 – 7.67 days). In contrast, the 11 patients carrying mutations at codon 268 of *pfcyt-b *presented with recrudescent infections on average 28.4 days after treatment (95% C.I. 23.9 – 32.9 days). Therefore, carriage of mutations in *pfcyt-b *is significantly associated with delayed recrudescent infections in these two studies of malaria patients presenting to European hospitals.

## Discussion

Atovaquone-proguanil (AP) has been widely adopted in the UK as a chemoprophylactic agent for travellers likely to be exposed to *P. falciparum *infections. AP is also widely employed as first-line treatment for cases of uncomplicated falciparum malaria in a number of London hospitals. Reports of AP treatment failure in the UK and Europe describe substitution of Tyr with Asn, Ser or Cys at codon 268 of the *pfcyt-b *gene in recrudescent parasites, typically three to five weeks after the initial malaria infection has been cleared by AP treatment [3]. Five further cases of *P. falciparum *recrudescence three to six weeks after initially successful AP treatment for uncomplicated malaria in travellers are described here. Parasites from one of these patients carried the Tyr-268-Ser mutant allele of *pfcyt-b *and the remaining four carried the Tyr-268-Cys allele. The 268-Cys allele has recently been reported in two French patients with *P. falciparum *malaria [5], but was first described in *cyt-b *genes of drug-selected *Plasmodium yoelii *isolated from atovaquone-treated mice [2]. This is the second report of this allele in human malaria infections.

In one case, patient 8, it was possible to compare the *pfcyt-b *sequence of recrudescent parasites with that of parasites present 26 days earlier. As previously observed [9], the appearance of parasites with the mutant *pfcyt-b *locus only among recrudescent parasites suggests that this mutation arose *de novo *in this individual due to AP selection. However, *pfcyt-b *mutations have been found among African parasite isolates without a history of atovaquone exposure [6], and it remains a possibility that such parasites occur at low prevalence in malaria endemic areas, and are occasionally selected by AP treatment of travellers in particular. Examination of mitochondrial sequences for evidence of selective sweeps around the mutant *pfcyt-b *loci, suggesting spread of a restricted number of mutant clones, would be instructive [10].

In contrast, three patients exhibiting persistent parasitaemia after 60 – 90 hours of AP treatment harboured parasites with wild-type *pfcyt-b *loci. The poor response to treatment in these patients infected by apparently atovaquone-sensitive parasites is consistent with the findings of Musset *et al *[5], who demonstrated low plasma levels of atovaquone, proguanil and cycloguanil (an active metabolite of proguanil) in four patients with slow-clearing infections immediately following AP treatment. Parasites isolated from these patients exhibited *in vitro *sensitivity to atovaquone, and did not carry mutations at codon 268 of the *pfcyt-b *locus. Malabsorption of atovaquone is thus one possible cause of reduced drug plasma levels and poor AP treatment response in the first few days following initiation of therapy. Drug levels were not determined for any of the patients described here, but such measurements would have strengthened the study.

Two travellers presenting in the UK with *P. falciparum *infection despite AP prophylaxis-use harboured parasites with a wild-type *pfcyt-b *locus. It is unclear whether the failure of prophylaxis in these two cases was due to poor absorption of atovaquone, sub-optimal adherence to the prophylaxis regimen or other unknown parasitological or host factors. Measurement of plasma levels of atovaquone in these patients may have helped to resolve these possibilities. A third UK traveller who used mefloquine prophylaxis for only part of her time at risk of malaria infection, patient 10, reported self-treatment with AP for fever while in Senegal. Thus the parasitaemia diagnosed in the UK is almost certainly a recrudescence of the first infection treated with AP some weeks earlier and so this case is consistent with the general observation that *pfcyt-b *mutations are associated with late recrudescent *P. falciparum *parasitaemia. The partial use of mefloquine prophylaxis in this particular case history, including an additional dose after return to the UK, one month before the appearance of recrudescent parasites, raises the possibility these parasites were also mefloquine resistant.

The conclusion that mutations at codon 268 of *pfcyt-b *are associated with delayed recrudescence of parasites after AP therapy is only partly in agreement with the findings of a large multicentre study, in which several cases of AP treatment failure were not associated with *pfcyt-b *mutations [4]. At least one of these cases represented a lack of response immediately after treatment, rather than a recrudescence. Four patients assessed by these authors experienced parasite recrudescence three to five weeks following AP treatment, but only one harboured parasites with the Tyr-268-Ser allele of *pfcyt-b*. However, Wichmann and colleagues used the RFLP method of Schwöebel *et al *[11] to identify *pfcyt-b *mutations. Examination of the respective recognition site sequences verified that, firstly, the restriction enzymes employed in this method, *Nsi*I and *Alw*NI, will not digest the Tyr-268-Cys allele found in three patients reported here and secondly, that this allele would be erroneously identified as a wild-type Tyr due to digestion only by the enzyme *SspI*. Therefore, it is possible that the remaining three individuals described by Wichman *et al *[4] as having recrudescent parasitaemia some weeks after AP treatment were harbouring the Tyr-268-Cys allele, which was not detected by the RFLP method. Although these authors state that mutations at other positions in the *pfcyt-b *gene were ruled out by sequencing a 716 bp PCR fragment, no data are shown to indicate whether or not the Tyr-268-Cys allele was or was not present. The results of the present study, and those of Musset *et al *[5], emphasize the importance of DNA sequencing approaches in identifying emerging, as opposed to established, drug resistance in malaria parasites.

The findings reported here can inform both treatment policy and advice given to prospective travellers to malaria endemic areas. Current data suggest that AP affords highly effective prophylaxis for endemic region travel, and is an efficacious treatment for uncomplicated cases of imported malaria. However studies with adequate follow-up of both treated cases and prophylaxis users are needed to verify these observations. Denominators for our study are difficult to estimate and, in the absence of active follow-up, some treatment failures may have presented for re-treatment elsewhere or not at all. Estimates from one of the hospitals participating in this study (St Thomas') are that AP alone was used to treat approximately 280 patients with confirmed *P. falciparum *malaria from Jan 2004 to December 2007. During this period only three apparent treatment failures have been confirmed as carrying parasites with *pfcyt-b *mutations, suggesting a resistance rate of approximately 1%. It is also worth noting that two of these cases occurred in non-immune patients. Bearing in mind that approximately 90% of falciparum malaria cases at St. Thomas' hospital affect semi-immune individuals who were born and brought up in endemic areas, this suggests that the acquired immunity to malaria enjoyed by many malaria patients may reduce the risk of occurrence of recrudescent infection associated with *pfcyt-b *mutations, as previously observed in studies of atovaquone monotherapy [1].

The results presented here also provide some support for the suggestion of Musset *et al *[5] that treatment failure after AP treatment may be caused by poor atovaquone bioavailability in some patients, and that slow clearance of parasites after AP treatment may be misinterpreted as treatment failure [4]. Preliminary results using quantitative SYBRgreen PCR in real-time, suggest that quinine treatment clears parasites approximately 40% faster than AP, and this delay in effect may also lead to some over-reporting of AP resistance *in vivo*.

## Conclusion

A series of *P. falciparum *isolates from malaria patients in the UK suspected of AP treatment (or prophylaxis) failure were tested for mutations in the *pfcyt-b *locus by direct DNA sequencing of PCR amplified products. Five patients with late, recrudescent treatment failure harboured mutations at position 268, and four of these were the recently described Tyr-268-Cys allele. This allele would not have been detected by restriction fragment analyses used in other studies. Three patients with apparent early treatment failure did not carry mutations, and may have failed to clear parasites due to low bioavailability of atovaquone. For purposes of drug resistance surveillance, DNA sequencing of the *pfcyt-b *locus is recommended in cases of late recrudescence of *P. falciparum *malaria, but may not be indicated where initial treatment response is delayed.

## Competing interests

The authors declare that they have no competing interests.

## Authors' contributions

CJS conceived the study, performed experiments, analysed data and wrote the first draft of the paper. ML contributed to study design, performed experiments, analysed data and contributed to writing the paper. NP, QF and GP contributed patient samples or reagents and contributed to writing the paper. MB performed experiments and analysed data. JLK and PLC contributed to study design and analysis, provided samples and edited the second draft of the paper.
